# Exploring Taurine's Potential in Alzheimer’s Treatment: A Comprehensive Review

**DOI:** 10.7759/cureus.60997

**Published:** 2024-05-24

**Authors:** Hira Aamer, Maha Tariq, Mehreen Yawar, Muhammad Shaheryar, Ali A Khan, Sadaf Iftikhar, Shahab Khan, Amjad Khan

**Affiliations:** 1 Department of Neurology, The King Edward Medical University, Lahore, PAK; 2 Department of Community Medicine, The King Edward Medical University, Lahore, PAK; 3 Department of Public Health and Nutrition, University of Haripur, Haripur, PAK; 4 Epidemiology and Public Health, University of Kentucky, Lexington, USA

**Keywords:** and experimental studies, cognitive impairment, neuroprotection, taurine, alzheimer's disease

## Abstract

Alzheimer’s disease (AD) stands as one of the predominant neurodegenerative disorders, often culminating in dementia. Taurine, an endogenous amino acid, holds pivotal regulatory functions within the physiological milieu. Emerging evidence suggests that taurine may confer protection against the onset and progression of AD through diverse mechanistic pathways.

This systematic review aims to comprehensively elucidate the multifaceted role of taurine in Alzheimer’s disease. The primary objective is to assess taurine's potential as a preventative and therapeutic intervention for Alzheimer's, based on studies from 2004 to 2022.

A rigorous search strategy was implemented, targeting English-language articles accessible in full text. Eligible studies were meticulously sourced from renowned databases including PubMed, PubMed Central, Science Direct, Cochrane Library, and Medline Plus. Inclusion criteria were limited to studies explicitly investigating the role of taurine in Alzheimer’s disease.

Our review encompasses a wealth of experimental studies conducted on murine models, collectively indicating taurine’s capacity to ameliorate symptomatic presentations of Alzheimer’s disease. Encouraged by these promising preclinical findings, the imperative for clinical trials in human subjects emerges. Taurine emerges as a prospective agent, offering potential mitigation of the cognitive and memory-related debility synonymous with Alzheimer’s disease.

This systematic review delineates a compelling body of evidence underscoring the putative neuroprotective role of taurine in Alzheimer’s disease. However, it is incumbent upon the scientific community to bridge the translational gap through robust clinical investigations. Such endeavors hold promise in revolutionizing the therapeutic landscape for individuals grappling with the formidable challenges posed by Alzheimer’s disease.

## Introduction and background

Alzheimer’s disease (AD) stands as a prevalent neurodegenerative disorder, representing a primary contributor to the onset of dementia. The pathogenesis of AD is characterized by the accumulation of neurofibrillary tangles and amyloid plaques within the brain parenchyma [1]. These misfolded proteins induce mitochondrial dysfunction, limiting energy supply to the brain and culminating in neuronal damage. Concurrently, the endoplasmic reticulum activates pathways leading to neuronal apoptosis. These mechanisms collectively precipitate neuronal death and the subsequent neurodegeneration seen in AD, which can manifest either as familial or sporadic in onset [2].

Taurine, a derivative of cysteine, is a naturally occurring sulfonic acid and the second most abundant amino acid produced in the central nervous system (CNS), surpassed only by glutamate [3]. Recognized for its neuroprotective properties, taurine plays a pivotal role in safeguarding nervous tissue against degenerative conditions. Its potential extends to the treatment of various neurological disorders, including AD, Huntington's chorea, Parkinson's, and stroke [3,4]. With multifaceted functions in the body, taurine regulates body temperature, stabilizes protein folding, mitigates inflammation, exerts antioxidative effects, maintains osmoregulation and calcium homeostasis, prevents apoptosis, and aids in CNS development [3,4]. Furthermore, it upholds the structural integrity of membrane proteins and exerts a neuro-modulatory influence by acting as a gamma-aminobutyric acid (GABA) and glycine agonist, thereby enhancing learning and memory processes [2].

Notably, elevated levels of taurine have been associated with a protective effect in the early stages of AD. Clinical trials supplementing AD patients with taurine have demonstrated potential benefits, which include a reduction in amyloid levels and cognitive symptom improvement. This review provides a comprehensive synthesis of existing studies, elucidating the prophylactic and therapeutic potential of taurine in combating neurodegenerative diseases, particularly AD. While experimental studies since 2004 have shown promise, primarily in murine models, the paucity of effective treatment options for AD underscores the urgency for exploration. Currently, no established treatment definitively halts or decelerates the progression of AD. This review posits that taurine holds promise for AD prophylaxis and may offer therapeutic benefits, as supported by extant experiments.

The objectives of this review are twofold: to scrutinize the neuroprotective effects of taurine in AD and to provide a comprehensive assessment of the studies investigating its impact and advantages in AD. Additionally, this review aims to delineate the mode of action of taurine in AD. It also explores its potential clinical applications, and ascertains its prophylactic and therapeutic efficacy in AD, with potential implications for future AD patients.

## Review

Methods

Eligibility Criteria

The review considered articles published in the English language between the years 2004-2022. Only original, accessible full-text articles that specifically investigated the role of taurine in AD were included.

Exclusion Criteria

Articles published outside of the stipulated time frame or in languages other than English were excluded. Additionally, preprints, newspaper articles, and book reviews were not considered. Studies focusing on neurological diseases other than AD were also excluded to maintain a specific focus on the function of taurine in AD.

Study Selection Process

The search for relevant articles was conducted across the following databases: PubMed, PubMed Central, Science Direct, Cochrane Library, and Medline Plus. The review was conducted by the Preferred Reporting Items for Systematic Reviews and Meta-Analyses (PRISMA) guidelines.

Search Strategy

In PubMed, we utilized the advanced search with the following query: (Taurine AND Neurodegenerative OR Alzheimer*). A filter was applied to restrict the search to articles published between 2004-2022.

Results and Screening

This initial search yielded 288 results, which were exported to an Excel sheet. Three independent researchers manually screened these based on abstracts to identify articles meeting our inclusion criteria. Eleven articles were deemed eligible, with two inaccessible. One article was initially included but subsequently excluded after full-text screening.

PubMed Central

A similar approach was employed for PubMed Central using the query (Taurine (Title) AND (Neurodegenerative (Abstract OR Alzheimer* Abstract), with the same date filter. Among the 14 results, two were duplicates and one was excluded after full-text review. Duplicates were removed manually.

Science Direct

In Science Direct, the search terms 'Taurine' and 'Alzheimer' were entered under the advanced search categories ‘Find articles with these terms’ and ‘Title, abstract or author specified keywords’, and articles were limited to those published between 2004-2022. This search returned 34 results, which were extracted into an Excel sheet through the Parse hub. One article met the inclusion criteria after manual review, and duplicates were removed.

Cochrane Library and Medline Plus

For the Cochrane Library, the search terms ‘Taurine AND (Alzheimer* OR Neurodegenerative)’ were entered under the search category ‘Title Abstract Keyword’ with a date filter of 2004-2022. Although 11 results were obtained, none met the eligibility criteria.

A final search was conducted in Medline Plus using the terms ‘Taurine’ and ‘Alzheimer’ for the years 2004-2022, which yielded zero relevant results.

Data Extraction

Each included article was independently reviewed by two researchers. Relevant data about the effect of taurine was manually extracted and discussed.

PRISMA Flow Diagram

**Figure 1:**
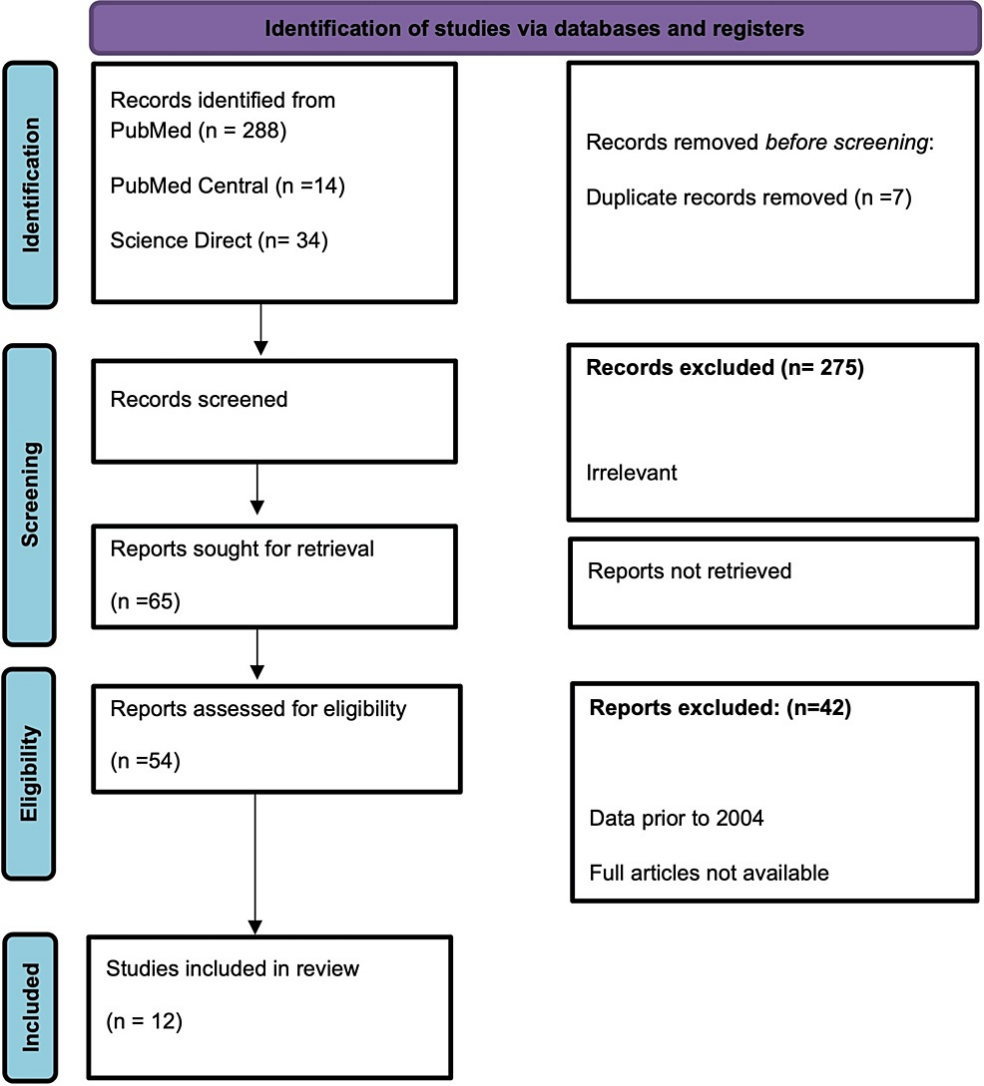
Preferred Reporting Items for Systematic Reviews and Meta-Analyses (PRISMA) Diagram.

PRISMA guidelines were strictly followed throughout the study and manuscript writing (Figure 1).

Epidemiological Data Source

The utilization of the Global Burden of Disease (GBD) 2019 (http://ghdx.healthdata.org/gbd-results-tool) analysis provides a comprehensive and up-to-date understanding of the epidemiology of AD and other dementias, spanning trends in incidence, prevalence, mortality, and disability-adjusted life years (DALYs) from 1990 to 2019 worldwide [5]. This ensures that our study is grounded in the most recent and globally recognized data, enhancing the reliability and relevance of our findings.

Results

Experimental Studies on Taurine in AD

All the included articles were experimental studies that aimed to assess the efficacy of taurine in improving symptoms of AD.

Epidemiology of AD

**Figure 2:**
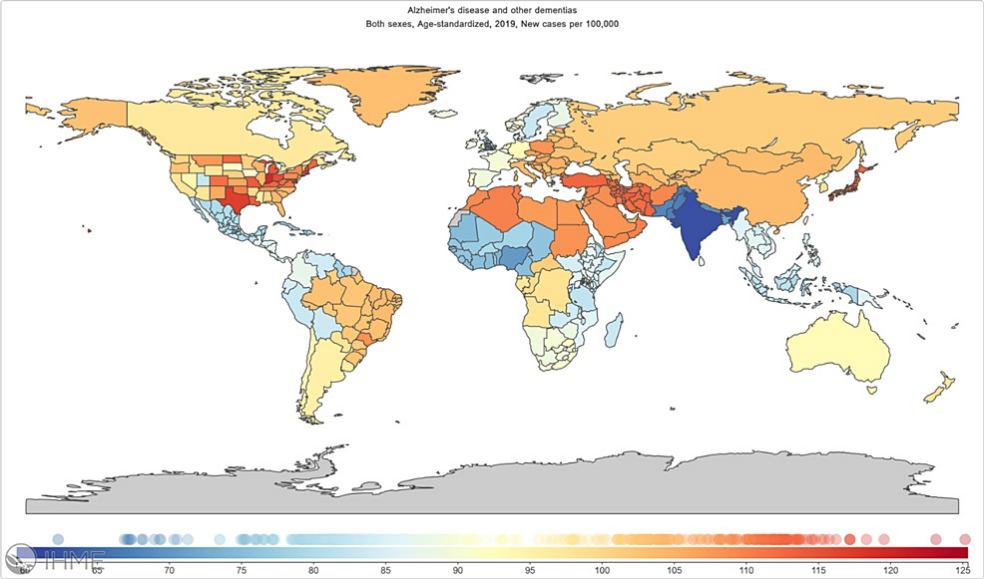
Distribution of the incidence of Alzheimer's disease and other dementias in 204 countries and territories from 1990 to 2019 by age standardized per 100,000 individuals of population (https://vizhub.healthdata.org/gbd-compare).

Dementia, tightly linked with the aging process, has witnessed a staggering increase in incidence (147.95%), prevalence (160.84%), and mortality (189.29%) rates worldwide [6]. It is projected that by 2050, the incidence of dementia will reach a staggering 152.8 million cases (Figure 2) [6].

**Figure 3:**
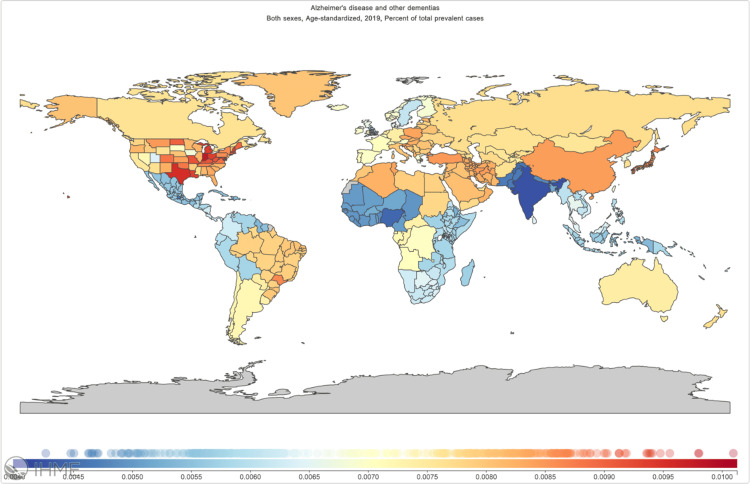
Distribution of the prevalence of Alzheimer's disease and other dementias in 204 countries and territories from 1990 to 2019 by age standardized per 100,000 individuals of population (https://vizhub.healthdata.org/gbd-compare).

Global prevalence and incidence are presented in Figure 3 trending an elevated increase for the last three decades. Addressing this global health challenge necessitates not only advances in therapeutic interventions but also a deeper exploration of potential neuroprotective agents like taurine.

**Figure 4:**
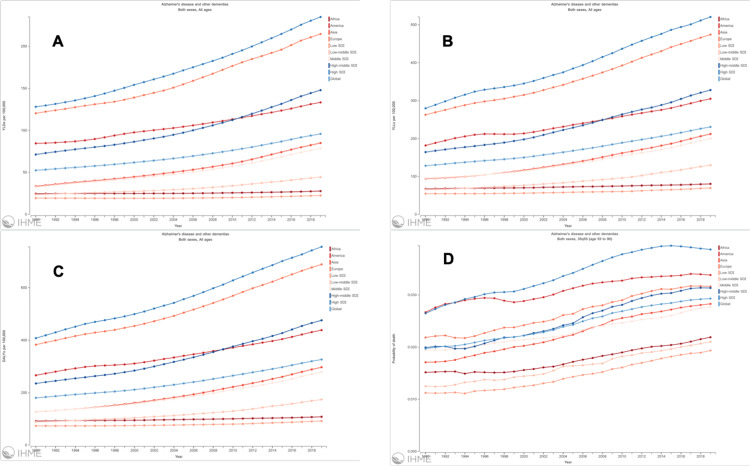
Distribution of YLDs, YLLs, DALYs per 100,000 individuals of population, and probability of death due to Alzheimer's disease and other dementias from 1990 to 2019 Globally and SDI regions. A. Years lived with disability (YLDs); B. Years of life lost (YLLs); C. Disability adjusted life years (DALYs); D. Probability of death globally and different sociodemographic index (SDI) as indicator-based regions (https://vizhub.healthdata.org/gbd-compare).

Global distribution of years lived with disability (YLDs), years of life lost (YLLs), and DALYs per 100,000 individuals of population and probability of death due to AD and other dementias from 1990 to 2019 globally and Socio-Demographic Index (SDI) regions are presented in detail in Figure 4.

Discussion

Effect of Taurine on Cognitive Function

In an experimental study, the researchers explored the therapeutic potential of taurine in the early stages of AD dementia using an adult APP/PS1 transgenic mouse model. The mice were administered taurine orally at a dose of 1000mg/kg/day for six weeks. The results showed that taurine supplementation led to improved behavioral performance in the APP/PS1 mice, as indicated by the Y maze test. Additionally, spatial memory in these mice improved significantly after six weeks of taurine treatment. The passive avoidance test further supported the positive impact of taurine on behavior and hippocampal memory. Notably, sandwich enzyme-linked immunosorbent assay (ELISA) of brain samples revealed a significant reduction in insoluble amyloid beta 42 levels in the cortex of taurine-treated mice [7]. These findings suggest that taurine may mitigate the damage caused by amyloid plaques and improve cognitive deficits.

A similar study employed a seven-week-old mouse model to assess the effects of taurine. Taurine was administered orally at a lower dose of 250mg/kg/day in this study. The results indicated improved spatial memory and enhanced hippocampal memory in the taurine-treated group, suggesting that lower doses of taurine may also have therapeutic benefits in AD [8].

While both studies demonstrated the cognitive benefits of taurine, they did not directly compare the efficacy of the two different doses. However, the second study implied that the lower and more practical dose of 250mg/kg/day might produce similar therapeutic results as the higher dose of 1000mg/kg/day, which could be promising for clinical applications [8].

Effect of Taurine on Amyloid Beta

In a study, the impact of taurine on AD mice was assessed using a glutamate Positron Emission Tomography (PET) scan [3]. Taurine was administered orally at a dose of 1000mg/kg/day based on the findings [7]. The results showed increased uptake of the PET tracer in the taurine-treated group compared to the AD group, indicating that taurine may inhibit the effects of amyloid beta oligomers, which are central to AD pathogenesis. However, there was no significant change in the concentration of amyloid beta plaques. These results suggest that taurine may have a therapeutic role in AD by targeting amyloid beta oligomers.

Another study investigated the impact of taurine on spatial learning, memory performance, and emotional learning. Rats were treated with a dose of 1000mg/kg/day of taurine for six weeks, along with intracerebroventricular injection of amyloid beta 1-42. While taurine supplementation did not significantly improve spatial learning and memory, it did enhance emotional learning and reduce anxiety-related behavior [9].

Prophylactic and Therapeutic Effects of Taurine

An experiment explored the prophylactic and curative effects of taurine on phosphorylated tau protein levels in rats' brains induced by scopolamine, mimicking AD-like symptoms. Taurine administered as pretreatment or treatment showed significant reductions in phosphorylated tau protein levels, with the highest dose of 100mg/kg/day exhibiting the most pronounced effect when used prophylactically. This suggests that taurine may be more effective as a prophylactic measure against AD-like mechanisms [10].

Another study revealed that taurine administration following streptozotocin (STZ) injection in rats improved cognition. Taurine at doses of 60 mg/kg and 120 mg/kg effectively mitigated cognitive impairment induced by STZ injection. These findings suggest that chronic taurine treatment can attenuate cognitive deficits in AD-like models [11].

Another experiment revealed that pretreatment with taurine resulted in improvement in cognitive function in one-year-old rats induced with STZ. Taurine exhibited a safeguarding effect against the detrimental impacts of STZ when administered prophylactically [12].

Neuroprotective Mechanisms of Taurine

Taurine has demonstrated neuroprotective properties in several ways. It has been shown to protect against mitochondrial dysfunction and neuronal death caused by amyloid beta [13]. Additionally, taurine has been observed to interfere with amyloid aggregation [14], further highlighting its potential therapeutic role in AD.

A study suggested that taurine's neuroprotective effects may be attributed to its ability to increase the vascular production of hydrogen sulfide (H2S), which in turn decreases amyloid beta levels and tau phosphorylation. Taurine was found to enhance the expression of enzymes involved in H2S production, contributing to its neuroprotective role [15].

Furthermore, taurine has been recognized as an agonist of GABA receptors [2]. Experimental studies have shown that taurine can protect neurons from the toxic effects of amyloid beta through GABA receptor activation. This GABAergic mechanism may play a crucial role in preventing and treating AD [16,17].

Neurological Disorders and Taurine

Beyond its potential in AD, taurine's neuroprotective properties hold promise for various neurological disorders. Taurine's role in neuromodulation, membrane stabilization, antioxidation, and apoptosis prevention makes it a candidate for treating a range of neurological conditions [2]. Targeting specific neurotransmitter receptors, such as GABA receptors, through medications like taurine may provide a novel approach to managing neurological disorders [17].

Role of Taurine in Brain Aging Protection

The presence of lower taurine levels in the brains, cerebrospinal fluid (CSF), and blood of AD patients highlights a potential connection between taurine and AD pathology [18-20]. Studies affirm that taurine plays a crucial neuroprotective role by reducing endoplasmic reticulum stress, preventing oxidative stress-induced apoptosis, and guarding against the neurotoxic effects of amyloid-beta [4,13,16,17].

Moreover, taurine's activation of GABA receptors presents a potential avenue for addressing neurological disorders characterized by GABAergic system dysfunction [21]. Experimental studies underscore taurine's efficacy in preventing neuronal death induced by amyloid-beta [16,17]. Furthermore, taurine's capacity to reduce neuronal susceptibility to excitotoxic damage offers a potential strategy for protecting against neurotoxicity induced by amyloid-beta and glutamate receptor agonists [17].

Taurine and Mitochondrial Function

Mitochondrial dysfunction is a critical component of AD pathology. Taurine, through its demonstrated role in preserving mitochondrial function, shows promise in mitigating the neurodegenerative processes associated with AD [13].

Taurine and Amyloid Aggregation

The ability of taurine to interfere with the aggregation of amyloid-beta, a hallmark of AD, represents a significant finding [14]. By reducing the accumulation of aggregated beta-amyloid, taurine presents a potential avenue for slowing the progression of the disease.

Taurine and Glutamate Receptors

Excitotoxicity, a process involving excessive activation of glutamate receptors, is implicated in the neurodegeneration seen in AD. Taurine's role in reducing neuronal susceptibility to excitotoxic damage holds promise for mitigating this aspect of the pathology of AD [17].

## Conclusions

The escalating global prevalence of dementia, particularly AD, necessitates a comprehensive approach to both understanding and treating this condition. This systematic review underscores the potential of taurine as a neuroprotective agent. Through its multifaceted mechanisms, including mitochondrial protection, interference with amyloid aggregation, and reduction of neuronal susceptibility to excitotoxicity, taurine emerges as a promising candidate for future therapeutic interventions. While these findings are promising, further rigorous clinical trials are imperative to validate and refine these observations. As taurine advances as a potential player in AD therapeutics, it offers a glimmer of hope in improving the lives of those affected by this devastating condition. Addressing existing gaps in knowledge through future research is crucial for fully understanding taurine's therapeutic potential in AD. Rigorous studies will help translate these promising findings into practical clinical applications.
